# Quinine as an Oxytocic

**Published:** 1875-12

**Authors:** J. C. Bishop

**Affiliations:** Middleport, Ohio


### QUININE AS AN OXYTOCIC.

BY J. C. BISHOP, M. D., MIDDLEPORT, OHIO.

*Read before the Ohio Valley Medical Society, April* 14, 1875.

It has been the custom for many years to administer to the parturient woman, under certain and peculiar conditions, medicines which were intended to promote delivery. The custom now prevails to such an extent, that when we assert that nineteen twentyeths of the more intelligent physicians of to-day habitually administer remedies for their oxytocic effects, under these certain restrictions, of course, we believe the assertion is none too sweeping, and yet the majority will hardly undertake to give a rational ex-

† Lime-water is probably the best.

planation of the physiological action of the drug so administered, nor why so many excellent practitioners obtain such diametrically opposite effects from the same drug, administered for the same purpose. Such however we know to be true. And without attempting to explain how, why, or where certain remedies do promote delivery, (which is comparatively "subjudice,") we offer as a meagre contribution to the evidence already adduced by writers, who claim for quinine some oxytocic properties, the imperfect report of two cases of labor, wherein this peculiar action of the drug was so positively manifest as to leave no doubt in our own mind that the effects attributed were actually due thereto. These cases have not been gotton up for the occasion, but actually occurred, and the attendants present will bear evidence that the effects were produced after the administration of the drug in question.

Case 1. Was called in the evening of Friday, January 29th, 1874, to attend Mrs. B., æt. 19, in her first confinement. Patient was medium size, nervous temperament and well developed. On my arrival, found her in bed, suffering the peculiar pains usual in the first stage of labor. A vaginal examination revealed the os uteri delating and dilatable, having already proceeded to about the size of a silver half dollar, while nothing abnormal was detected in the presenting part of the fœtus. As time passed the os became fully dilated, the protruding tense amniotic membranes were ruptured, and to my surprise, the finger encountered the left ear of the child, which must have assumed that position whilst the liquor amnii was still escaping. By manipulation both externally and internally, the presentation was converted into one of the face, and after much trouble the chin was brought under the symphisis pubis, as offering the most eligible position for delivery. The pains, which from the moment of the rupture of the membranes had been gradually failing in efficiency (about two and one half hours having elapsed,) became weak, extremely short, and utterly powerless so far as could be observed. Here we should have given ergot, if we had been fortunate enough to have had any, (and it generally goes into our obstetric armamentiere,) but we could get none. Here was a typical case in which to try quinine, which had seemed to act well in the hands of some; consequently, we at once caused the patient to take six grains of the drug, and sat by to note the result. In from twenty to thirty minutes the pains began to come on with more violence and efficeiency; thirty minutes after the first dose, another of similar quantity was administered; the contractions kept increasing in efficeincy, and in one hour after the first dose had been given, the patient was delivered of a large male child, weighing ten pounds. She made a rapid and satisfactory recovery, and at this date both mother and child are doing well.

CASE 2. Mrs. C., æt. 24, nervous habit, was in labor with her second child; was well developed, and of fine physique. Was summoned early Tuesday morning, Febuary 16th, 1875; on arrival found that the first stage of labor had just terminated, the membranes having ruptured just before my arrival; an examination per vaginam revealed a first vertex presentation, while the funis prolapsed slightly; her first labor had been easy and natural. The cord was replaced in the usual manner, and kept as far beyond pressure as possible. A short time after the rupture of the membranes, the contractions, which had heretofore been unusually severe, began to lessen in severity and efficiency, until they appeared almost entirely inert, and exhibited none of the characters of expulsive pains; were irregular in character, the interval between them being long. As in the former case we administered six grains of quinine, which had the remarkable effect of reestablishing the uterine effort, of increasing the length and severity of the contractions, and labor was terminated rapidly and satisfactorily, and patient and child are both doing well.

The effect of the drug administered in these two cases, it seems tolerably positive, was to increase the efficiency of the uterine contractions when they were utterly powerless so far as relates to expulsive force. That no other aids were given, such as irritation of the os etc., would at once preclude the idea of mechanically increasing the pains. Therefore, the effects following the administration of the drug alone in both cases wherein the condition was somewhat similar, together with the characters of the pain, leaves no doubt in our own mind but that they were the legitimate result of the remedy. We have tried the same agent in other cases with like happy results. It is not at all presumed in this paper to claim that quinine is capable of generating the uterine effort *de novo*, or that its use in pregnant women will produce abortion; on the contrary the evidence seems to be strongly in opposition to this doctrine. That the oxytocic effect of this agent should offer any pretext for its being withheld in pregnant women, wherein it would otherwise be indicated, seems to us to be a delusion, and the idea cannot be supported by the array of clinical evidence necessary to render it either just or generous.

